# ACTivity as medicine In Oncology for Head and Neck (ACTIOHN): a feasibility study investigating a patient-centred approach to exercise for head and neck cancer patients

**DOI:** 10.3389/fonc.2025.1525512

**Published:** 2025-07-11

**Authors:** Joanne M. Patterson, Mary G. Cherry, Andrew Levy, Simon N. Rogers, Rachel C. Brooker, Valerie M. Bryant, Steven Lane, Michael M. Nugent, Ruth Price, Andrew G. Schache, Jennifer A. Kirton, Bridget Young, Debra Fisher, Adrian W. Midgely

**Affiliations:** ^1^ Liverpool Head and Neck Centre, School of Allied Healthcare Professionals and Nursing, University of Liverpool, Liverpool, United Kingdom; ^2^ Institute of Population Health, University of Liverpool, Liverpool, United Kingdom; ^3^ Department of Psychology, Edge Hill University, Ormskirk, United Kingdom; ^4^ Arrowe Park Hospital, Wirral University Teaching Hospital National Health Service (NHS) Foundation Trust, Liverpool, United Kingdom; ^5^ Head and Neck Oncology, The Clatterbridge Cancer Centre National Health Service (NHS) Foundation Trust, Liverpool, United Kingdom; ^6^ Cancer of Head and Neck Group Experience (CHANGE) Patient and Public Involvement Group, Sunderland Royal Hospital, Sunderland, United Kingdom; ^7^ Institute of Infection, Veterinary, and Ecological Sciences, University of Liverpool, Liverpool, United Kingdom; ^8^ Oral and Maxillofacial Surgery, Sunderland Royal Hospital, South Tyneside and Sunderland National Health Service (NHS) Foundation Trust, Sunderland, United Kingdom; ^9^ Liverpool Head and Neck Centre, Liverpool University Hospitals National Health Service (NHS) Foundation Trust, Liverpool, United Kingdom; ^10^ Liverpool Head and Neck Centre, Department of Molecular and Clinical Cancer Medicine, University of Liverpool, Liverpool, United Kingdom; ^11^ Department of Sport and Physical Activity, Edge Hill University, Ormskirk, United Kingdom

**Keywords:** head and neck cancer, rehabilitation, prehabilitation, physical exercise, feasibility, personalisation, remote delivery

## Abstract

**Objective:**

To determine the feasibility and acceptability of integrating a remote, personalised, collaborative, and flexible exercise programme into the head and neck cancer (HNC) care pathway.

**Design:**

A single arm mixed-methods feasibility study across two UK NHS hospitals.

**Procedure:**

Eligible HNC patients (aged ≥ 16 years old, treated with curative intent and classified as low/medium risk according to an exercise risk stratification tool) were invited to participate between diagnosis and 8 weeks post-treatment. Patients treated with palliative intent and those identified as high risk on an exercise risk stratification tool were excluded. Following initial assessment, Cancer Exercise Specialists (CESs) and patients collaboratively devised a personalised exercise programme based on a needs analysis, preferences and goals, and informed by physical activity cancer guidelines and theory. CESs were trained in behaviour change techniques. The intervention was flexible and delivered remotely across 8 weeks, with weekly meetings and texts, and an exercise maintenance plan agreed in the final session.

**Outcomes:**

Eligibility, recruitment, retention and exercise adherence were primary outcomes. Quantitative outcomes included quality-of-life, fatigue and physical activity questionnaires and physical fitness tests. A qualitative sub-study explored patients’ and healthcare professionals’ (HCPs’) views on feasibility and acceptability.

**Results:**

98% of patients screened were eligible; 107 patients were approached, and 76 consented (71%). Most (43%) were recruited pre-treatment. Three quarters were male and just over half had oropharyngeal cancer. Thirteen patients (17.1%) were withdrawn due to ill-health. Twenty-three (30.3%) patients dropped out, 13 after assessment but before the intervention, and ten during the intervention. Forty patients (52.6%) completed the intervention. Three quarters of exercise sessions were completed as prescribed. Patient interviews found the flexible, personalised approach valuable. Those not identifying as an ‘exerciser’ found the intervention more difficult to understand. The need for more education for both HCPs and patients regarding the benefits of exercise and its ‘fit’ within the HNC pathway was highlighted.

**Conclusion:**

This is a feasible and acceptable intervention, but some adjustments are required, to improve acceptability, recruitment processes, retention and adherence, before examining effectiveness in a definitive trial.

**Clinical Trial Registration:**

https://www.isrctn.com/ISRCTN82505455, identifier ISRCTN82505455.

## Introduction

Rates of head and neck cancer (HNC) are rising globally, with approximately 660,000 new cases per year. Risk factors include significant smoking and alcohol consumption and Human papillomavirus (HPV) (1). The increase in rates has been attributed to HPV-associated oropharyngeal cancer, where patients are younger at diagnosis with improved survival outcomes, and thus living longer with chronic treatment side effects. However, the incidence of HNC is highest in those >70 years, a patient population with considerable co-morbidities, significant weight loss and higher levels of frailty (2). HNC incidence rates are substantially higher in the most deprived quintile compared with the least (3). HNC patients from poor socio-economic backgrounds typically have low levels of support (4, 5), and suboptimal health literacy, which limits their ability to acquire and understand healthcare information and access services (3, 6). Up to 60% of patients are diagnosed with advanced disease at presentation, stage IV being the most common stage for oral and oropharyngeal cancer (7, 8).

HNC treatment is often aggressive and multi-modal, leading to numerous acute and long-term side effects that are often debilitating. Patients frequently experience challenges such as difficulty swallowing, weight loss and malnutrition, difficulty speaking and breathing, pain, fatigue and the psychological impact of altered appearance. These issues frequently occur in parallel and have a significant negative impact on people’s lives, wellbeing and quality of life (QoL). There is evidence to suggest that physical exercise can help to reduce cancer treatment-related side-effects, the negative impact of co-morbidities, distress and mortality rates, whilst improving mood and QoL (9–12). The majority of cancer exercise research is conducted in breast, colon and lung cancer groups, with data for HNC patients severely underrepresented (13). Generalising findings to HNC is problematic given considerable differences contrasting HNC patients from other cancer survivors, with important implications for exercise prescription (14); such as inherently lower levels of physical activity pre-treatment, poorer cardio-pulmonary fitness and a substantial symptom-burden as previously mentioned (14–16).

Preliminary evidence suggests that physical exercise for HNC can reduce pain (17) and fatigue (17–19); while improving strength (17–20) and cardiorespiratory fitness (17, 18). However, there is weak evidence supporting its impact on body composition, body mass index, or QoL (17, 18). The safety profile of physical exercise interventions appears acceptable, with minimal adverse events reported (18). To date, most HNC studies have described exercise interventions led by physiotherapists (an often-scarce resource (21)) within a specialist centralised cancer treatment centre (22). The content of interventions focuses on exercise type and intensity, though there is insubstantial evidence to definitively determine which exercise might offer the most benefit. A combination of aerobic and resistance training may offer greater benefits than a single type of full-body exercise during radiotherapy treatment (18, 23). The optimal timing of initiating an exercise programme, however, remains unclear (20), as some studies initiate exercise before treatment, while others focus solely on the post-treatment period.

The feasibility and acceptability of an exercise intervention in HNC remains in an exploratory stage. Reported recruitment rates range from 36-72%, with sample sizes ranging from 40–60 participants (15, 19, 24). Available evidence is limited by variable retention and adherence rates with some as low as 54% for a pre-chemoradiotherapy intervention (19). The World Health Organisation acknowledges that there are substantial barriers to patients following treatment plans, such as socio-economic, patient-related, disease-related and factors relating to the health care team (25). In HNC, reported barriers to exercise are often linked to disease and treatment-specific symptoms, particularly pain (general and head and neck-related), fatigue and difficulties with eating and drinking (26). Patient concerns regarding safety and fear of doing harm also hamper participation. A lack of time, interest, motivation, financial restrictions, ability to travel and access to facilities are additional constraints (27). Healthcare professionals (HCPs) can have an important role in encouraging HNC patients to exercise through information provision on the benefits of exercise, and advice on overcoming HNC specific problems e.g. exercising with a feeding tube, and on-going support to encourage adherence (26). Furthermore, perceived physical and mental benefits, gaining a sense of control and self-efficacy can motivate patients to continue with an exercise programme (26).

Behavioural strategies used to address physical activity barriers and encourage adherence are an important but often under-reported part of HNC exercise interventions. A systematic review, using behaviour change theory to understand adherence to HNC swallowing exercises, cited the following strategies to promote adherence: 1) providing instruction on how to perform the behaviour, 2) setting behavioural goals, 3) action planning, 4) offering support, and 5) tracking activity with guidance from a trusted source, such as a healthcare professional (28). These strategies may be useful to consider for HNC physical exercise interventions.

In summary, there is limited evidence supporting the recommendation that physical exercise enhances physical and mental health, as well as QoL, in HNC (17). Retention and adherence are problematic within this group. Reviews suggest that a tailored and personalised approach, which addresses both facilitators and barriers, may improve adherence and retention for exercise interventions. This study sought to address these challenges by exploring the feasibility and acceptability of integrating a remotely delivered, fully personalised, collaborative, and flexible approach for prescribing and delivering exercise programmes into the usual HNC care pathway.

## Materials and methods

### Study design

The full study protocol has been published (29) and is summarised below, in accordance with the CONSORT extension for pilot and feasibility studies reporting checklist (30). This was a two-centre single arm mixed-methods feasibility and acceptability study. No significant changes were made to the methods reported in our study protocol (29) following study commencement. A favourable ethical opinion was granted by West of Scotland Research Ethics Service (22/WS/0058). A minor amendment was approved in December 2023, modifying the qualitative sub-study recruitment procedures. The study was conducted in accordance with the Declaration of Helsinki. The study was registered on 23 August 2022 (ISRCTN82505455).

### Study objectives

To determine:

1. Eligibility, recruitment, retention, and exercise adherence.
2. Frequency, intensity, time, and type of exercise, and timing of the start of the exercise programme, based on patient clinical need and personal preferences.
3. Intervention and participant fidelity.
4. Suitability of outcome measures and provide data to inform a sample size calculation for a definitive randomised controlled trial (RCT).
5. HNC patients’ and HCPs’ views on acceptability, intervention components, processes, and feasibility of integrating into the usual care pathway.

### Participants and settings

The patient eligibility criteria are summarised in **Table 1**.

**Table 1:** Patient eligibility criteria.

Inclusion	Exclusion
≥ 16 years old able to provide informed consent diagnosed with HNC and being treated with curative intent between the time of diagnosis and up to 8 weeks post-treatment classified as low or medium risk according to an exercise risk stratification tool [Morgan and Irwin (31)]	high risk according to the exercise risk stratification tool any unstable or uncontrolled medical condition associated with increased risk during exercise HNC patients treated with palliative intent

Patients were consecutively recruited from two regional National Health Service HNC centres; site 1 in North West England, site 2 North East England. Patients who initially declined participation were asked for consent to be re-approached for the study after starting their cancer treatment or again at their first post-treatment consultation.

### Assessments

Patient reported measures and physical activity tests were collected by specialist physiotherapists, conducted at the hospital sites, before and after the intervention. These were secondary outcomes and informed the intervention. Patient reported measures were 1) Multidimensional Fatigue Symptom Inventory - Short Form (MFSI-SF), assesses of fatigue across five domains: general fatigue, physical fatigue, emotional fatigue, mental fatigue, and vigour 2) Short-Form 36 Health Survey Questionnaire (SF-36) assesses limitations in: physical activities because of health problems; social activities because of physical or emotional problems; usual role activities because of physical health problems; bodily pain; general mental health; usual role activities because of emotional problems; vitality (energy and fatigue); and general health perceptions 3) International Physical Activity Questionnaire short form (IPAQ-SF) assesses physical activity domains of: job-related; transportation; housework, house maintenance, caring for family; recreation, sport, and leisure-time. Also assesses time spent sitting; physical fitness tests were 30-Second Chair Stand Test, 30-Second Arm Curl Test, 8-Foot Up-and-Go Test, 6-Minute Walk Test, and shoulder and cervical range of movement (32–37) (see **Supplementary File 1**).

### ACTIOHN intervention

The intervention was a personalised and flexible 8-week exercise programme, delivered remotely (telephone or video-call) by cancer exercise specialists [CESs; a definition of a CES and their qualifications are stated in the study protocol paper (29)]. Assessment data, together with any HNC-specific physiotherapy advice, were transferred to a CES prior to the CES contacting the patient. Programmes were co-designed by the patient participant and the CES. At the first meeting, the CES conducted a remote patient needs analysis and developed a personalised plan. Frequency, intensity, time, and type of exercise were informed by baseline fitness assessments, the participant’s exercise history, exercise preferences, perceived barriers to exercise, and personal goals. Exercise selection and modifications were also based on addressing any HNC-specific issues. Each programme was set within a framework of current physical activity guidelines for cancer survivors (38–40) including the frequency, intensity, time, type, volume, and progression (FITT-VP) of the programme (41); see **Supplementary File 2** for an example.

The CESs were given written guidance on how to prescribe the exercises, including factors to consider when personalising the exercise, the principles of using a collaborative approach, how to integrate flexibility (autoregulation) into the exercise programme, and the details of the FITT framework for each exercise modality. Each participant’s exercise preferences took precedence. Accordingly, concurrent aerobic and resistance training was encouraged but participants were permitted to only perform one of these types of training if preferred. Resistance training options included resistance band exercises and body weight exercises. Free weight and resistance machine exercises were incorporated if the participant had access to the relevant equipment. Flexibility exercises were included, guided by the physiotherapists and baseline flexibility assessments. Balance exercises were only included if the participant had any balance issues.

The CES met with the patient a second time to go through the planned programme. Before beginning the programme, each participant was given a ‘Physiotherapy Advice and Information’ sheet with information on recognising signs and symptoms for which the patient should seek immediate medical attention during the intervention (see section on adverse events and monitoring). A selection of free, accessible and locally available or online options, partially guided by prior research was offered to help patients choose their preferred form and setting for exercise, while also inviting them to suggest alternatives based on personal preferences and past successes in maintaining an exercise routine (29). Participants were given access to Physitrack^®^ software (Physitrack PLC, UK) containing videos on how to safely and effectively perform the exercises they had been prescribed.

The CES conducted weekly individual consultations by video or telephone call with each participant during the 8-wk programme. Each consultation lasted approximately 30 minutes and was logged via written meeting notes stored on Microsoft Teams. The meeting content was guided by a written protocol document. Consultations included patient education, resolution of perceived exercise barriers, support to promote exercise adherence based on behaviour change theory, checking safe and effective exercise technique, discussing safety issues, and encouraging accurate and timely completion of the participant’s logbook. Any adverse symptoms were discussed and the CES sought advice from the referring physiotherapist where this was deemed appropriate. The CES sent two texts per week to each participant containing a motivational message to promote programme adherence, and a reminder to complete logbooks.

Prior to recruitment, CESs were trained in techniques to support patients’ behavioural change. The training comprised two educational workshops, each lasting 2-hours and were delivered remotely by a chartered psychologist accredited by the British Psychological Society (42). Grounded within the self-determination theory approach (43), the workshops incorporated motivational interviewing skills (44) and behaviour change techniques (45). In addition to the behaviour change support training, CESs received fortnightly intervention supervision with fidelity checks from a certified exercise physiologist.

Further behavioural support entailed the use of peer stories, developed with our public and patient involvement (PPI) group, to encourage physical activity engagement for HNC patient participants. Our PPI lead gathered HNC physical activity stories among the broader PPI group. These stories were then carefully curated, ensuring they cover a range of experiences and highlight practical strategies for overcoming common barriers. The main goal of using real-life peer stories was to empower HNC patients to recognise the benefits of being active during and after their treatment. The stories were shared through an ACTIOHN brochure that was disseminated to HNC patients by the physiotherapists at the first assessment.

### Qualitative sub-study

Patients and staff were invited to participate in an interview. Patients were purposively sampled to cover a range of possible experiences, including according to their age, gender, cancer type and stage, treatment, Index of Multiple Deprivation (IMD) decile, whether they completed, declined or withdrew from the programme, activity status and timing of approach (pre-, during or post-treatment). Patients were selected and contacted at differing time points (during and after the programme) and spanned those with full to limited intervention compliance. HCPs involved in their care, including the CESs employed to design and deliver the activity programmes, were also included in the study. HCPs and CESs were sampled according to their role and degree of involvement with ACTIOHN. Recruitment methods are detailed in the study protocol (29).

Patient semi-structured interviews focused on the intervention acceptability, study processes and applicability of outcome measures, while staff interviews covered assessments and programme delivery, usability of the study materials and intervention tools, as well as the acceptability of the mode and timing of intervention delivery. Interviews were audio recorded and transcribed verbatim. The analysis followed the principles of the constant comparative method (46) and interpretive reflexive thematic analysis (47), considering both latent and manifest aspects of the data. Systematic data coding was performed, and exceptional case analysis was discussed within the research team. To enhance rigour, data were triangulated with quantitative data to enrich findings and interpretation. Procedurally, the “following a thread” framework for triangulation and integrative analysis of qualitative and quantitative data was adopted (48), with data regularly discussed and triangulated at project management groups meetings. Qualitative data were reported in accordance with relevant guidance (49).

### Outcome measures

1. Eligibility, recruitment and retention rate.
2. Adherence: number of: i) sessions completed as prescribed; ii) missed sessions; iii) sessions modified before the session started; iv) sessions modified during the session; and v) sessions terminated early.
3. Frequency, intensity, time (duration), and type of exercise and the starting point of the exercise programme.
4. Physical activity tests, patient-reported measures, and their completion rate.
5. HNC patients’ and HCPs’ views on feasibility and acceptability.

**Figure 1:**
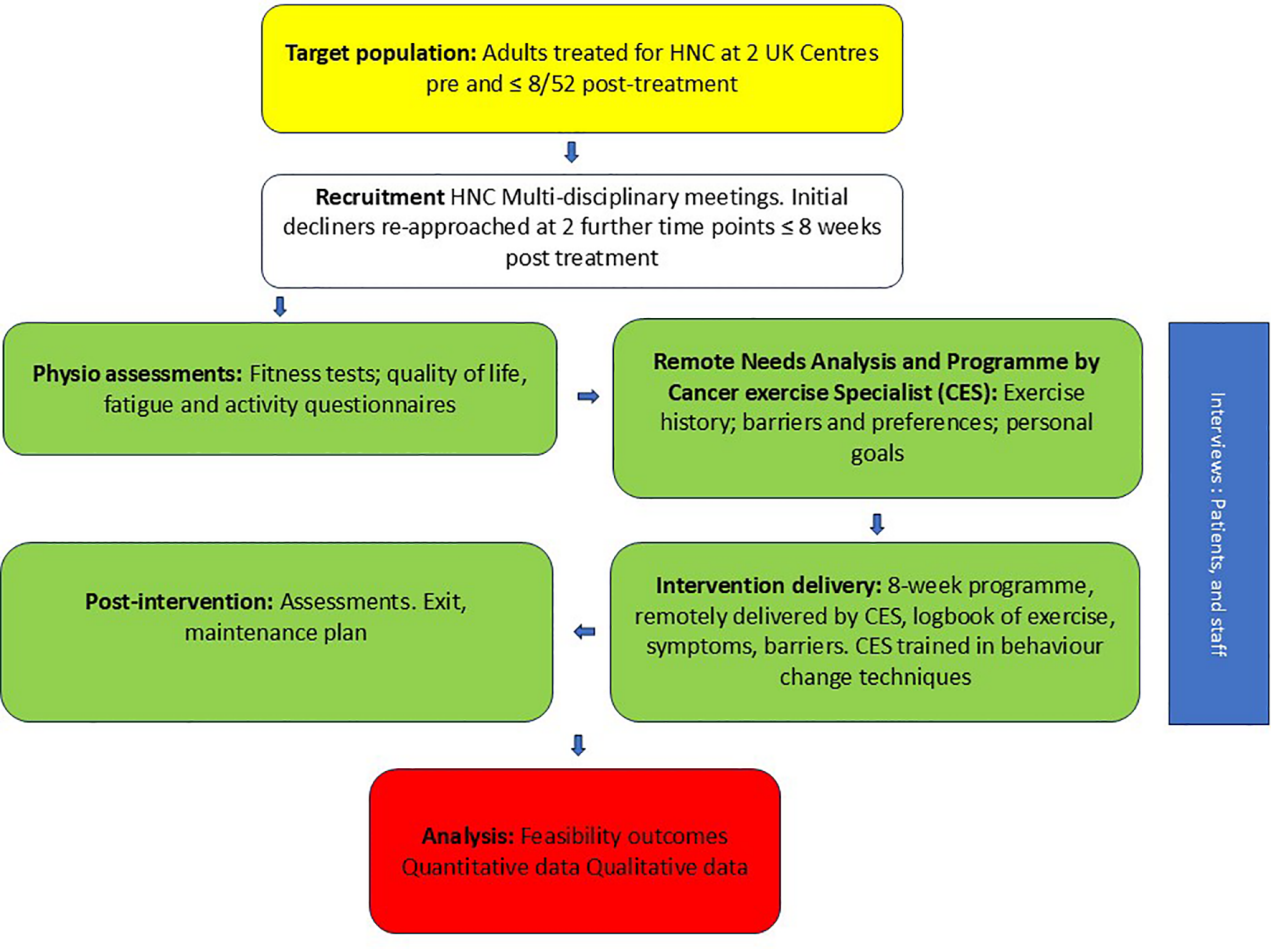
Flow diagram of recruitment, intervention and follow-up.

A summary of the study and a schedule of events are provided in **Figure 1**, **Table 2**.

**Table 2:** Schedule of events.

Procedure / assessment	Screening and approach	Pre-intervention	During intervention	Post-intervention
Morgan and Irwin risk stratification tool	X			
Consent and registration	X			
Fitness assessments		X		X
Patient reported measures		X		X
Patient needs and preference analysis		X		
Physiotherapy Advice and Information sheet		X		
CES remote support meetings with patients			X	
CES motivational texts			X	
Exercise logbook completion			X	
Safety and adverse events monitoring			X	X
Exercise maintenance plan				X
Patient interviews			X	X
Staff interviews			X	X

### Sample size

The study aimed to recruit 70 patients, and to retain a minimum of 42 patients on completion based on a conservative estimated retention rate of 60% (50–53). The target sample size was determined pragmatically using guidelines for feasibility studies recommending a sample size of between 24 and 50 (54–56). We planned to interview approximately 20 patients and 20 HCPs (N = 40).

### Data analyses

To assess trial feasibility, we calculated the proportion of eligible patients who consented to participate, recruitment rate and retention until the end of the intervention. Patient flow from screening, consent and intervention completion was summarised using a consort diagram. Availability of outcome data at each time point was summarised. As this was a feasibility study, no formal hypothesis tests were undertaken. Baseline and follow-up demographic and outcome data were summarised using standard summary statistics such as means, medians, and percentages, along with corresponding 95% confidence intervals. No p-values were reported as the study was not powered to test for differences in the assessment measures. Data for patients who completed the intervention and non-completers (including those withdrawn by the study or patients who dropped out) are presented. All analyses were undertaken using the STATA software package (Version 17, StataCorp LLC, Texas, USA).

### Success criteria

The feasibility of this study progressing to a larger scale trial, was determined by the pre-specified traffic light progression criteria (29, 57) and qualitative sub-study findings (see **Supplementary File 3**).

### Safety and adverse events monitoring

Patients classified as high risk according to the risk stratification tool, along with clinical judgement regarding suitability for enrolment onto the study via discussions at multidisciplinary team meetings were excluded from participation.

Potential adverse events identified for the intervention included: musculoskeletal injuries e.g. muscle strains, ligamental sprains, and joint injuries; fall-related injuries; unexplained limb or face weakness or loss of sensation; cardiovascular event; breathing difficulties; hypoglycaemia/hyperglycaemia; confusion or disorientation; loss of vision; syncope; seizure; severe delayed onset muscle soreness; dislodged feeding tube or leak. The Physiotherapy advice sheet provided a list of potential adverse signs and symptoms in connection with the intervention. They were asked to seek immediate, emergency help, should they experience cardio-respiratory symptoms i.e. sudden unexplained breathlessness at rest, central chest pain, irregular heart rate or palpitations; neurological symptoms i.e. sudden loss of sensation/unexplained weakness/pins and needles in a limb/face, seizures or collapse, confusion, loss of vision/headaches; bony abnormalities i.e. sudden or severe pain in the back, ribs or long bones, new deformity of a limb, swollen leg especially if hot to touch/red, or other symptoms (appearing pale, dizziness when standing up, unexplained bruising or nose bleeds, unexplained persistent severe pain, fever, overall feeling of weakness and general tiredness, unexplained lump/swelling). Patients were advised that if their medical condition changed or they were unsure whether to continue exercising, to contact the physiotherapist or the research nurses. Patients continued to receive treatment as usual which included multi-disciplinary weekly reviews during radiotherapy and regular follow-ups post-treatment.

The physiotherapists were the main contact for reporting adverse symptoms; out-of-hours contact details were provided. The CESs were responsible for monitoring patients during their weekly meetings, including enquiring about any changes in health status, medications, and symptoms. If concerns were raised, the CES contacted the physiotherapist for further guidance. Any adverse events and reactions were contemporaneously recorded by patients in their logbook during the intervention. Any adverse event up until seven days following the end of participation in the intervention was recorded at the research site. Any serious adverse events occurring from baseline up until the last follow-up was reported to the research lead within 24 hours (recorded on a programme-specific form) and reported to the NHS Health Research Authority within 15 days. A serious adverse event was defined as (a) results in death; b) life-threatening; c) requires hospitalisation or prolongation of existing hospitalisation; d) results in persistent or significant disability or incapacity; f) is otherwise considered medically significant by the research lead.

### Public and patient involvement

A PPI group of six members with lived experience of HNC was convened for the study. They met on six occasions. Their activities included the development of patient support materials, approach and consent processes, patient information sheets, dissemination plans, qualitative methods training and interview results discussions, as summarised in **Supplementary File 4**.

## Results

### Eligibility, recruitment, and retention

**Figure 2:**
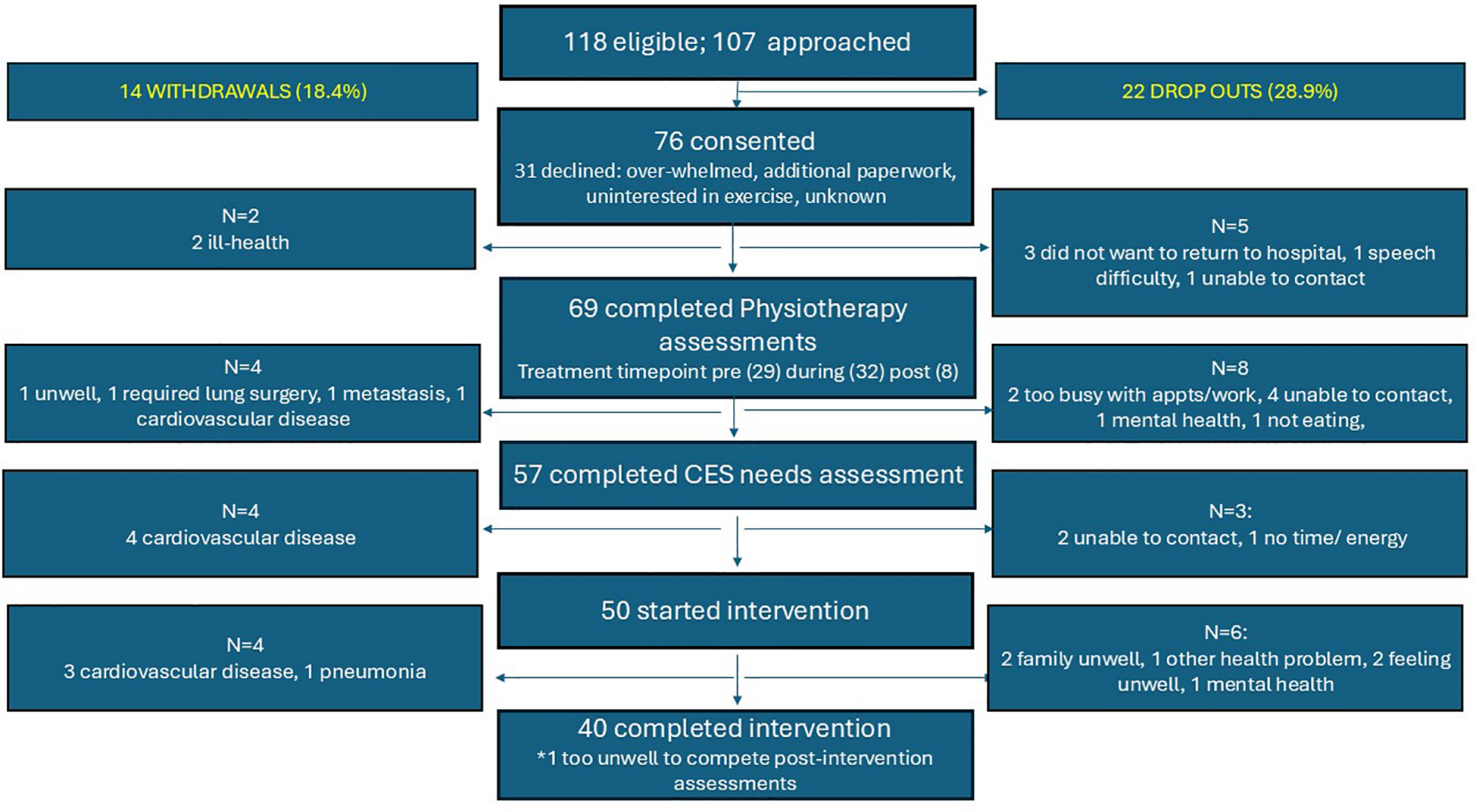
Reasons for declining participation, withdrawals, and drop-outs. CES, Cancer Exercise Specialist.

Site 1 opened to recruitment during September 2022, followed by site 2 in January 2023. Recruitment closed in August 2023, resulting in a recruitment period of 12 and 8 months for Sites 1 and 2 respectively. Eighty-three patients were screened at Site 1 and 37 patients at Site 2. In total, 118 of these 120 patients were eligible for the study. Of these, 107 (90.6%) patients were approached for ACTIOHN, and 76 (71.0%) agreed to participate (Site 1: n = 56; Site 2: n= 20 patients). One patient agreed to be interviewed but declined participation in the intervention. Seventy-five patients agreed to participate on the first recruitment approach, one patient agreed on the second approach. Fourteen patients (18.4%) were withdrawn due to ill-health (two before physiotherapy assessment, four following physiotherapy assessment, four following the remote needs assessment and four during the intervention). Reasons included cardiovascular disease, pneumonia, significant wound infection, and further surgery for a lung lesion, rendering them ineligible to continue in the study according to the risk stratification tool. Twenty-two (28.9%) patients dropped out, sixteen after physiotherapy and remote needs assessment but before the intervention (reasons were travel to hospital, unable to contact, speech difficulties, mental health), and six during the intervention (reasons were poor mental health, feeling unwell and other caring responsibilities). These occurred after week one (n=1), after week two (n=4) and after week six (n=1). In total, 40 patients completed the intervention. A summary of eligibility, participation, and withdrawals is shown in **Figure 2**.

### Patient characteristics and treatment

Demographic data and treatment information was available for 70 patients. Data was missing for six patients who consented but did not participate in the intervention (three withdrawals, three dropouts) (see **Table 3**). The average age was 61 years, the majority were male (77.1%) and co-habiting (81.2%). A range of deprivation scores were recorded. Just over half of the patients had an oropharyngeal tumour (n=42), 31 (74%) of these were HPV positive. Fifty-nine patients had surgery, 15 with free flap reconstruction. Forty-six had adjuvant treatment (35 had radiotherapy, 11 had chemoradiotherapy) and 13 had no adjuvant treatment. At the time of recruitment, no patients had a laryngectomy or tracheostomy, and ten patients had a gastrostomy and three had a nasogastric tube.

**Table 3:** Baseline demographic data.

Data		Site 1	Site 2	Completers	Non-completers	Total
N		56	20	40	36	76
Age (yr)	Mean (st dev)	62.6 (10.6)	54.7(11.7)	60.6 (10.8)	61.6 (11.1)	61.0 (10.9)
	Range	(39, 82)	(34, 74)	(39,82)	(34,80)	(34, 82)
	Missing	4	2	0	6	6
Sex n(%)	Male	38 (73.1)	16 (88.9)	29 (72.5)	25 (83.3)	54 (77.1)
	Female	14 (26.9)	2 (11.1)	11 (27.5)	5 (16.7)	16 (22.9)
	Missing	4	2	0	6	6
Marital status n(%)	Single	12 (12.7)	4 (25.0)	8 (20.0)	8 (27.6)	16 (23.5)
	Married	37 (71.2)	10 (62.5)	31 (77.5)	16 (55.2)	47 (69.1)
	Widowed	2 (3.8)	0	1 (2.5)	1 (4.4)	2 (2.9)
	Divorced	1 (1.8)	0	0	1 (4.4)	1 (1.5)
	Other	0	2 (12.5)	0	2 (8.8)	2 (2.9)
	Missing	4	4	0	8	8
Ethnicity n(%)	White	52 (100.0)	15 (87.5)	39 (97.5)	28 (96.5)	67 (97.0)
	Asian/Asian British	0	1 (6.3)	1 (2.5)	0	1 (1.5)
	Black/African Caribbean/	0	1 (6.3)	0	1 (3.5)	1 (1.5)
	Black British	0	0	0	0	0
	Missing	4	3	0	7	7
Living arrangements n(%)	Alone	11 (21.2)	1 (6.2)	5 (12.8)	7 (24.1)	12 (18.8)
	With others	40 (76.8)	16 (93.8)	34 (87.2)	22 (75.9)	56 (81.2)
	Missing	5	3	1	7	8
Deprivation decile n(%)	1 (least deprived)	15 (29.4)	5 (31.3)	12 (30.9)	8 (28.6)	20 (29.4)
	2	8 (15.7)	3 (18.8)	5 (12.8)	6 (21.4)	11 (16.2)
	3	11 (21.6)	3 (18.8)	10 (25.6)	4 (14.4)	14 (20.6)
	4	6 (11.7)	4 (25.1)	5 (12.8)	5 (17.8)	10 (16.2)
	5 (most deprived)	11 (21.6)	1 (6.3)	7 (17.9)	5 (17.8)	12 (17.6)
	Missing	5	4	1	8	9
BMI (kg/m ^2^)	Mean (st. dev)	27.34 (6.4)	29.07 (4.47)	27.3 (5.4)	28.5 (6.9)	27.78 (6.06)
	Range	(17.3, 51.2)	(24.2, 39.4)	(20.0,44.6)	(17.3,51.2)	(17.30, 51.17)
	Missing	6	2	1	7	8
Smoking status n(%)	Non-smoker	26 (50.0)	5 (33.3)	22 (56.4)	9 (32.1)	31 (46.3)
	Current	8 (15.4)	0	3 (7.7)	5 (17.9)	8 (11.9)
	Ex-smoker	18 (34.6)	10 (66.6)	14 (35.9)	14 (50.0)	28 (41.8)
	Missing	4	5	1	8	9
Alcohol status n(%)	Never	8 (15.4)	3 (20.0)	6 (15.8)	5 (17.8)	11 (16.2)
	Current	36 (69.2)	9 (60.0)	26 (68.4)	19 (67.9)	45 (66.2)
	Ex-drinker	7 (13.5)	3 (20.0)	6 (15.8)	4 (14.3)	10 (14.7)
	Missing	5	5	2	8	10
Tumour site n(%)	Oral cavity	14 (26.9)	8 (44.4)	12 (30.0)	10 (33.4)	22 (31.4)
	Oropharynx	33 (63.5)	9 (50.0)	25 (62.5)	17 (56.7)	42 (60.0)
	Larynx	3 (5.8)	0	1 (2.5)	2 (6.6)	3 (4.3)
	Nasal cavity	1 (1.9)	0	0	1 (3.3)	1 (1.5)
	Salivary gland	1 (1.9)	1 (5.6)	2 (5.0)	0	2 (2.8)
	Missing	4	2	0	6	6
Tumour stage n(%)	1	16 (30.8)	9 (52.9)	17 (42.5)	8 (27.6)	25 (36.2)
	2	21 (40.4)	3 (17.7)	13 (32.5)	11 (37.9)	24 (34.8)
	3	3 (5.8)	1 (5.9)	2 (5.0)	2 (6.9)	4 (5.8)
	4	12 (23.1)	4 (23.5)	8 (20.0)	8 (27.6)	16 (23.2)
	Missing	4	3	0	7	7
Tumour Node n(%)	0	16 (30.8)	6 (37.5)	9 (22.5)	13 (44.8)	22 (31.9)
	1	4 (7.6)	8 (43.8)	8 (20.0)	4 (13.8)	12 (17.4)
	2	30 (57.6)	3 (18.7)	22 (55.0)	11 (37.9)	33 (47.8)
	3	2 (3.8)	0	1 (2.5)	1 (3.5)	2 (2.9)
	Missing	4	3	0	7	7
Treatment n(%)	Surgery alone	8 (15.4)	5 (29.4)	5 (12.5)	8 (27.6)	13 (18.9)
	Surgery + adjuvant RT/CRT	36 (69.3)	10 (58.8)	29 (72.5)	17 (58.6)	46 (66.6)
	Primary RT	2 (3.8)	0	1 (2.5)	1 (3.5)	2 (2.9)
	Primary CRT	6 (11.5)	2 (11.8)	5 (12.5)	3 (10.3)	8 (11.6)
	Missing	4	3	0	7	7

RT, radiotherapy; CRT, chemoradiotherapy.

The majority of patients were recruited pre-treatment (42.1%) or post-surgery but before adjuvant radiotherapy, i.e. during treatment (46.3%), with just 11.6% following treatment. No serious adverse events associated with the intervention were reported.

### Exercise adherence

Of the 40 completers, 37 returned their completed logbook. There were 1238 exercises sessions prescribed for these 37 participants. A total of 1083 (87.5%) of these 1238 prescribed exercises sessions were performed, 147 (11.9%) were not engaged with, and 8 (0.6%) had missing logbook entries so it was not possible to discern whether these exercise sessions were performed. For the 1083 documented performed exercise sessions, 63 had missing logbook entries as to whether they were completed as prescribed. For the remaining 1020 exercise sessions, 854 (83.7%) were completed as prescribed, 100 (9.8%) were modified by the participant before the participant began exercising, 17 (1.7%) were modified by the participant during the exercise session, and for 49 (4.8%) exercise sessions the participant terminated the exercise session before completing it.

For the 313 exercise sessions that were documented as either not completed or not completed as prescribed, reasons were given by participants for 228 of these exercise sessions. Fatigue was the most common reason and the only reason for non-compliance for 96 (30.7%) of these 313 exercise sessions, or in combination with other symptoms for 34 (10.9%) exercise sessions. Feeling sick was the second most common reason and the only reason for 31 (9.9%) exercise sessions and in combination with other reasons for another 32 (10.2%) exercise sessions. Pain was reported as the only reason for 26 (8.4%) of the 313 exercise sessions and in combination with other reasons for another 12 (3.8%) exercise sessions. Other reasons were too busy (n = 9), poor weather (n = 7), issues with dry mouth, mouth secretions, and swallowing difficulties (n = 5), holiday period (n = 5), concerns about not eating enough and weight loss (n = 5), hospital appointment (n = 3), performed alternative (non-prescribed) exercise (n = 2), feeling light-headed (n = 2), temporal proximity of treatment to prescribed exercise session (n = 1), admitted to hospital (n = 1), fell asleep (n = 1) and physiotherapist advice (n = 1). There were 12 instances of exercise interruptions, defined as missing three or more consecutive exercise sessions, by 11 (29.7%) of the participants that completed the 8-week exercise programme and returned their completed logbook. The median (range) number of exercises sessions missed during these exercise interruptions was 5 (3 to 22).

Symptoms that participants perceived as being caused by the exercise were reported for 27 exercise sessions. Symptoms were pain and/or stiffness (n = 18), fatigue (n = 4), feeling sick/queasy (n = 2), dizziness (n = 1), dry/sore throat (n = 1), and tingling in the shoulder (n = 1).

### Exercise programme characteristics

Median (interquartile range; IQR) exercise frequency was 3 (2) times per week. Of the 1083 documented exercise sessions performed, 405 (37.4%) included only aerobic exercise, 315 (29.1%) only resistance exercise, and 363 (33.5%) included aerobic and resistance exercise. Median (IQR) aerobic exercise intensity was a rating of perceived exertion of 12.5 (1.0) and median (IQR) exercise duration was 35 (31) minutes. The type of aerobic exercise was reported for 757 of the exercise sessions that included aerobic exercise. Walking was the most common type with 71.1% of exercises sessions consisting of walking as the only type of aerobic exercise performed in the exercise session. Other types performed on their own were cycling (10.4%), exercise video (8.8%), running (4.2%), and swimming (1.1%). Combinations of types of aerobic exercise were performed in 4.3% of exercise sessions. The median (IQR) number of sets performed per exercise session for the resistance training was 14 (9), with a median (IQR) number or repetitions per set of 15 (3). The type of resistance training was reported for 631 exercise sessions. A combination of resistance bands and body weight exercises was the most common (70.5%). Others were combined resistance bands, body weight exercises, and free weights (14.4%), body weight exercises and free weights (4.3%), body weight exercises only (3.5%), resistance bands and free weights (3.3%), free weights only (2.7%), and resistance bands only (1.3%). Examples of HNC-specific adjustments to programmes include modifying resistance bands for patients with limited grip following a forearm flap reconstruction; for patients with a fibula flap the focus was initially on seated strength; for those with a gastrostomy passive abdominal exercises were avoided.

### Intervention and participant fidelity

Intervention fidelity was high, including full delivery of the behaviour change training to the CESs in accordance with the protocol, writing of exercise programmes within the exercise prescription framework written for the study, and maintenance of logs written by the CESs (i.e. notes of weekly support meetings between the CESs and the patients, and dates the CESs sent weekly texts to patients). Of the required weekly support meetings, 98.5% were conducted and 98.6% of the weekly support texts were sent by the CESs. The main reason for not meeting or sending texts was the Christmas vacation period. Regarding participant fidelity, there were 224 (18.1%) prescribed exercise sessions where the participants failed to provide one of more pieces of information regarding the exercise performed. Examples included not stating the aerobic exercise intensity, not stating the number of repetitions performed for one or more sets of resistance training exercises, or not confirming whether the exercise session was performed as prescribed. Only two exercise sessions were performed by participants that significantly deviated from what was prescribed. These both involved going for a long walk with other people rather than the prescribed exercise session.

### Outcome measures

A total of 69 MFSI-SF, SF36, IPAQ-SF questionnaires and physical fitness test batteries were completed at baseline, during the physiotherapy assessment (see **Supplementary File 5**). One participant completed the intervention but was too unwell to complete the measures at follow-up, leaving 39 post intervention questionnaires.

#### Fatigue: MFSI-SF questionnaire

**Figure 3:**
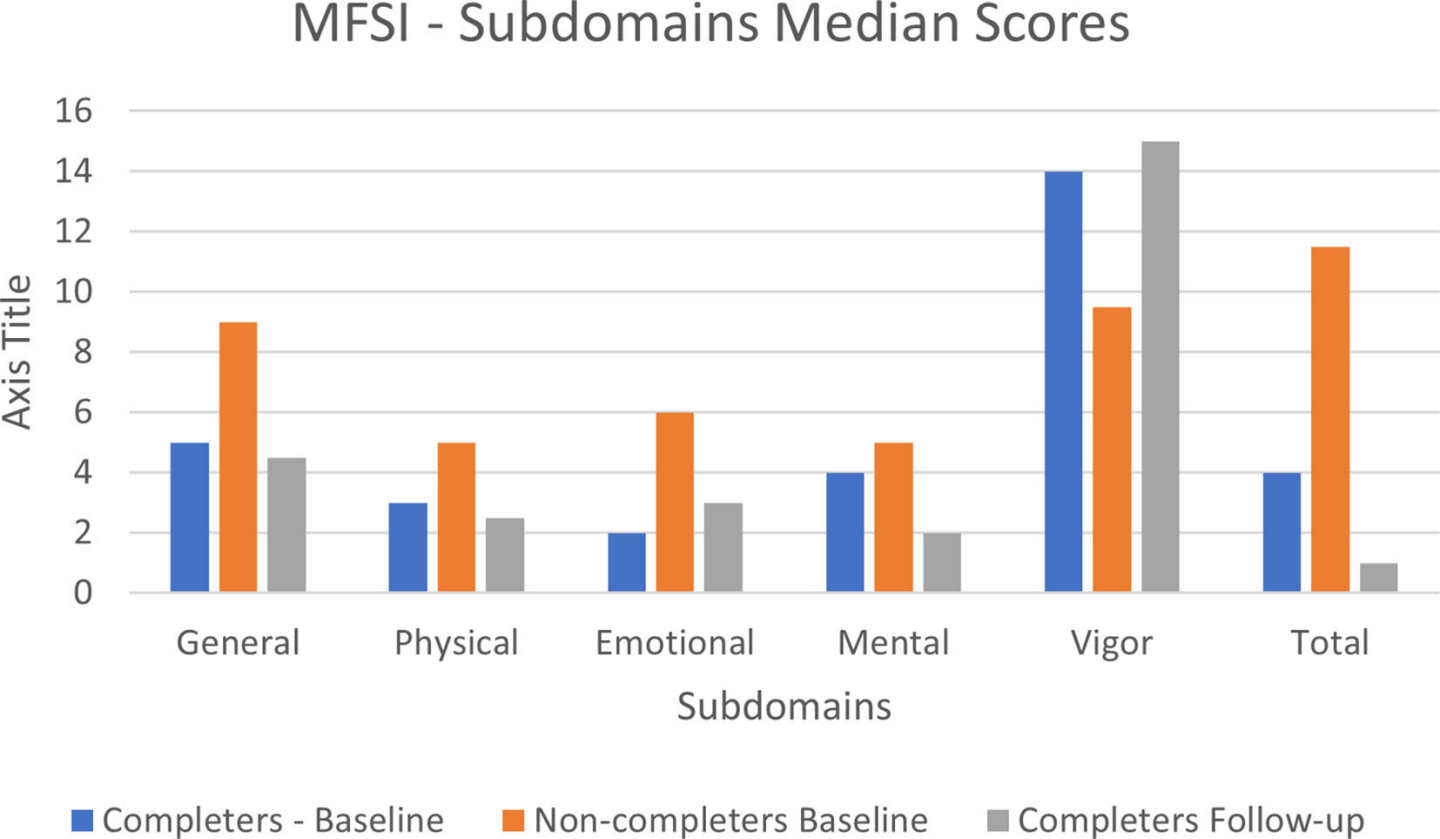
MFSI subdomains median score at baseline and follow-up.

Overall episodes of missing data were small, with most items having no missing values. The largest number of missing observations for a single item was 3, related to ‘feeling upset’. For subscales 1 to 4 (general, physical, emotional and mental fatigue) the non-completers had higher median values (i.e. more fatigue) than the completers at baseline. For the fifth subscale (vigor), the median value was higher for the completers (i.e. more vigor) compared with the non-completers. Overall, the total median score was higher in the non-completers group compared to the completers group (see **Supplementary Files 5**, **6**). There was very little change in MFSI-SF median scores between baseline and follow-up, with minimal missing data with only four items having missing observations (see **Figure 3**).

#### Health-related quality of life: SF-36

**Figure 4:**
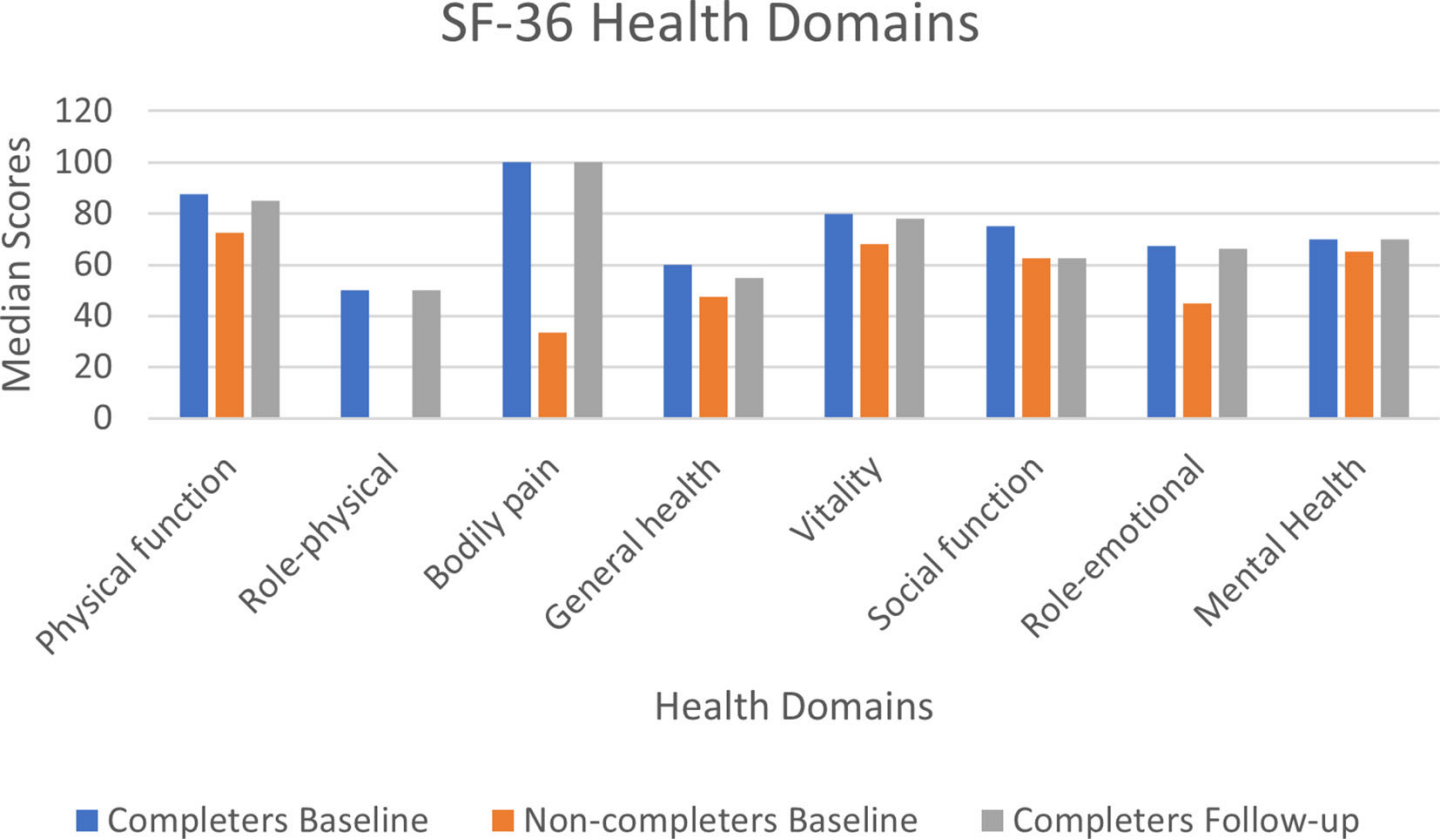
SF-36 health domains median scores at baseline and follow-up.

At baseline, there were minimal missing values. Item 3 (4 missing values) was the only item with more than two missing observations at baseline. At follow-up no item had more than a missing single observation. At baseline, median scores were higher (i.e. better) in the completers group compared to the non-completers for all health domains (see **Supplementary Files 5**, **6**). Similar median scores were obtained between baseline and follow-up for the 39 completers (see **Figure 4**).

#### Physical activity levels: IPAQ-SF

There were very few missing items for the IPAQ (4 at baseline, 2 at follow-up). Overall, 48/65 reported no vigorous physical exercises at baseline. This was split between the two groups, 23 in the non-completers group and 25 in the completer group. Thirty-one patients (19 non-completers and 12 completers) reported no moderate physical exercise. Of those who completed the questionnaire at follow-up, 18/37 reported no vigorous physical exercise and 11 reported no moderate physical exercise (see **Table 4**).

**Table 4:** Percentages for how many patients were engaging in low, moderate, and vigorous intensity physical activity pre and post intervention.

	Pre N=56	Post N =39
MET-min/wk median(IQR)	1194 (3321)	2514 (37.15)
range	(0, 14,666)	(0, 9278)
Exercise N(%) Low	23 (37.3)	7 (17.9)
Moderate	11 (15.9)	12 (30.8)
Vigorous	22 (31.9)	19 (48.7)

MET, metabolic equivalent of task.

#### Physical fitness assessments

All 69 participants were able to complete the chair stand test. Nine (four in the non-completer group and five in the completer group) were unable to do the arm curl test. This was due to it being too soon after surgery with free flap reconstruction. One participant failed to complete the 8 feet up and go test, due to fatigue. There was one missing value for the 6-minute walk test. For all four tests, there was little difference in median values between completers and non-completers at baseline, or between baseline and follow-up measurements (see **Supplementary File 7**).

For the shoulder movement assessments, there were minimal missing values (<4), these were due to post surgery pain. The majority had full flexion and abduction at baseline. Among the completers, at least 42.5% retained shoulder movements and 32.5% improved compared with baseline function.

The cervical measurements showed a larger improvement in movement with between 11 and 18 participants reporting an improvement in the six movements being measured. Those who reported a reduction in movement varied between 0 and 2, except for right rotation which had 5 participants reporting a reduction in movement.

### Qualitative sub-study: HNC patients’ and staff views

Eighteen patients (including one who declined participation, one who dropped out and two who were withdrawn due to cardiovascular disease) and 12 HCPs (two oncologists, two CESs, one physiotherapist, two research nurses, one speech and language therapist, two dieticians, one ear nose and throat consultant and one clinical nurse specialist) were interviewed. Six HCPs worked at Site 1, four at Site 2, and the remaining CESs worked across both sites. Five had been directly involved in ACTIOHN; the remainder were familiar with the study but had not directly recruited to it or been involved in its delivery.

Most patients self-identified as active and appreciated the benefits that a personalised physical activity programme could bring to their recovery. Some participants, particularly those who did not identify as active, initially found it difficult to understand the rationale behind the programme and study, as well as the extent of personalisation of the intervention. Once enrolled, patients valued the flexible, personalised nature of the programme. Communication between staff and patients was integral to continued engagement, particularly for patients whose understanding of the programme was poor. Practical issues (e.g. use of logbook, fit of programme around treatment side-effects) impacted negatively on delivery, adherence and acceptability. Specifically, most patients reflected that it was unrealistic or burdensome to expect patients to be active whilst going through HNC treatment, feeling that the best time to start a programme would likely be before treatment or as early as possible into treatment (so that they had begun to benefit from the routine and activity prior to onset of side-effects). If a programme took place during treatment rather than post-treatment, participants felt that pauses in the programme would be needed to allow people to take a sustained break if required. Patients benefitted from the programme in a plethora of ways; they enjoyed having an alternative focus during cancer treatment and recovery, were able to see tangible changes in their activity and fitness levels post-intervention and reported that the programme had positively impacted on their mood and motivation. Most reported a desire to continue with activity post-programme.

Staff reported divergent views regarding the appropriateness and value of the exercise programme for HNC patients and highlighted the need for more education regarding the benefits of the programme and its ‘fit’ with HNC treatment to aid implementation and their own understanding of the programme. Additional training needs were identified for HCPs, particularly the CESs (related to HNC-specific needs and adaptations such as treatment side-effects and working with patients with feeding tubes).

Patients and HCPs suggested improvements to the programme and study including: i) considering multiple ways of giving and receiving information to and from patients, such as digitising resources and outcome measures ii) refining the timing of the programme and/or permitting pauses in the programme to allow patients to gain maximum benefit from it; iii) providing patients with clear explanations of the rationale for the study, in accessible ways (e.g. verbally; videos; written materials), taking account of prior knowledge, activity levels, literacy and health literacy; iv) ensuring that all staff working with HNC patients are fully trained in the benefits and risks of activity, particularly those delivering the intervention; and v) ensuring that language is appropriate and consistent (e.g. use of the term activity rather than exercise). These are summarised in **Supplementary File 8**, alongside illustrative quotations.

## Discussion

This study reports positive outcomes of a clinical study of a patient-centred approach to exercise for HNC patients, integrating a remotely delivered, fully personalised, collaborative, and flexible approach for prescribing and delivering exercise programmes into the usual care pathway. To our knowledge, this is the first UK study to do so. This demonstrated that: 1) only a small number of patients were missed at site recruitment clinics and there was excellent uptake by eligible patients; 2) a high level of adherence to the exercise programmes; 3) patient drop-out was greater than expected, and although published recommendations indicate the number of completions was sufficient to estimate a suitable sample size for a full clinical trial, a sample size calculation was not conducted since patients were recruited across a range of timepoints across their cancer treatment pathway; 4) a high level of intervention fidelity, but issues with participant fidelity relating to incomplete logbook entries; 5) patients, HCPs, and the CESs expressed strong support for the study and intervention, although a need for further clarifications; and 6) a success criteria outcome of amber (see **Supplementary File 3**), indicating progression to an RCT is appropriate but with minor changes to the protocol.

### Recruitment, retention, and exercise adherence

The literature indicated a sample size of 40 would be sufficient to estimate an effect size for a full trial. Target recruitment was 70 eligible patients with an estimated retention rate of 60%. ACTIOHN had a broad eligibility criterion, including pre- or post- surgical +/- non-surgical treated patients. Our retention rate was slightly lower than planned (52%). Most patients who left the study, did so before the intervention began. Both HCPs and patients reported that balancing multiple appointments and information made participation difficult. Flexibility in clinical appointments may enable patients to better organise their time to accommodate regular activity. However, HNC care pathways are complex requiring input from multiple agencies; there is a strong driver to meet cancer time-to-treatment targets, in addition to patient desire to begin treatment quickly, reducing scheduling flexibility. For some, understanding the intervention at the outset was difficult, particularly for those unaccustomed to exercising. Fully personalising patient information in addition to the intervention may reduce drop out at this stage. The sample reflected typical HNC demographics, i.e. predominantly white males, with oral or oropharyngeal cancer. The full range of socio-economic status was represented in the sample.

Fourteen patients had to be withdrawn due to physical ill-health, before and during the intervention. Comorbidities such as cardiovascular problems and neuropathy are frequently cited barriers to participation (27). Approximately one third of HNC patients having chemoradiotherapy have unscheduled hospital admissions during their treatment (58). Further consideration is needed regarding the minimum level of activity/exercise for those people who are unwell, as they are unlikely to meet physical activity guidelines for cancer survivors. Accordingly, the median exercise frequency in the present study was 3 times per week, indicating that many of the participants did not meet current guidelines. An important principle stated in the guidelines, however, are that cancer survivors should avoid inactivity (38) and the input of clinical expertise (in accordance with the principles of evidence-based practice (59)), is an important consideration in helping to establish an appropriate amount of exercise. Autoregulation is another, albeit under researched strategy that has great potential to help deal with the issue of patients struggling to fulfil prescribed exercise due to symptoms relating to cancer and its treatment. Autoregulation involves patients modifying prescribed exercise sessions based on how they are feeling on the day and is recommended in the Exercise and Sports Science Australia position statement for exercise medicine in cancer management (40). Notably, almost 10% of exercise sessions in the present study were modified by participants before they started exercising, which was mostly due to cancer and cancer treatment-related symptoms.

Attrition was most likely to occur in weeks 1–3 of the programme. Qualitative data suggest that even for those patients that remained on the programme, engagement with the planned exercises was challenging during cancer treatment. General cancer treatment-related symptoms such as fatigue and pain are frequently cited as barriers to physical activity (27). These symptoms are known to be higher in HNC compared to other cancer types (60, 61). Specific HNC side-effects such as having a dry mouth and throat further impede participation (27). Treatment side-effects are a major barrier to remaining active and should be managed accordingly. Future studies may consider permitting breaks from the programme to accommodate periods of ill-health due to treatment side-effects, resuming in an incremental manner.

In the present study, participants performed 87.5% of the prescribed exercise sessions, 83.7% of which were completed as prescribed with no modifications or early session termination. There are no widely accepted definitions on how to interpret the level of exercise adherence; however, one study regarded participants as being “successful” at adhering to their prescribed exercise programme if they engaged in at least two-thirds of their prescribed exercise sessions (62). This was based on research showing significant improvements in functional capacity and markers of health from participating in at least two out of three prescribed exercise sessions per week. Other exercise intervention studies involving people with HNC during cancer treatment reported exercise adherence rates of 45.2-93.1% (15, 19, 24, 52, 63–65). Plausible explanations for the differences in adherence rates between studies include differences in the volume of exercise prescribed, the mode of delivery (e.g. home-based versus supervised centre-based), and the degree of personalisation. A major criticism of exercise oncology research is that a ‘one size fits all’ approach to the design and delivery of exercise programmes has largely been employed (66). A high level of personalisation and input from the patient during the exercise prescription stage is likely to be particularly important, given that physical activity levels are enhanced by a sense of power and control (26). Notably, many participants in the present study reported enjoying the highly personalised approach and tailoring throughout.

We attempted to address barriers to exercise associated with some previous studies by incorporating a highly personalised, collaborative, and flexible approach to exercise prescription, with remote delivery to negate the burden of travelling to and from a venue. Our findings support challenges identified by other studies, such as management of treatment side-effects and lack of understanding of the benefits of the intervention and what it involves (26, 27), which if addressed should improve adherence in future studies. Walking was the most common type of aerobic exercise in the present study and was the only type performed in 71.1% of the exercise sessions where aerobic exercise was advised. This is consistent with a survey of 430 people living with or beyond HNC, where 73% of respondents stated that walking was their preferred exercise option (67). Poor weather is perceived as a barrier to walking (68) and, therefore, might have been expected to negatively impact exercise adherence. Poor weather was documented as a reason not to adhere to an exercise session for only three exercise sessions, despite the study spanning the four seasons of the year.

### Outcome measures

As this was a feasibility study, there was no proposed primary outcome measure stipulated. Completion rates for all measures and assessments were high, passing the *a priori* progression threshold. Using these data to inform a sample size calculation was problematic. Patients were recruited at different timepoints during their cancer treatment, i.e. before, during and following treatment, when the expected direction of outcome for our selected measures was variable. However, our candidate measures enabled a comparison with other published literature. For the fatigue scale, published norms suggest a mean total score of 7.92 (69), which compares favourably with patients’ baseline measures (median score 7), although a large range was noted. A minimally clinically important difference of between 4.5-10.79 has been reported, although this was derived using data from an Asian breast cancer cohort (70). Those that completed the intervention had better emotional fatigue and total scores at baseline than the non-completers, indicating that our sample may be positively skewed towards patients with less fatigue at the outset. In ACTIOHN, the completers had better vigor and total scores (>4.5) at the end of the intervention. Their median scores appear lower (i.e. less fatigue) than reported following other physical activity interventions in HNC using the same scale such as Tai Chi (32.36, SD. 11.12) (71), and resistance exercises (43.4, SD 8.7) (72), although direct comparisons are limited by our study which focused on feasibility, not effectiveness.

Fatigue is the most common unmet need in HNC survivorship, with our previous work demonstrating a high prevalence of clinically relevant fatigue amongst HNC patients, affecting 45% at 4 months post-treatment, dropping to just under a third at 12 months (60). Being female, a current smoker, having advanced HPV negative disease, comorbidities and multimodal treatment are predictors of worse fatigue (73, 74). However, this symptom is often overlooked by clinicians due to the complex and multifactorial symptom profile of this group (75). Physical activity may be one way of attenuating fatigue.

The SF-36 was collected as a QoL measure. At baseline, the completers had better QoL scores than those reported by the non-completers. However, physical functioning scores were higher for all participants (median 75, IQR 45) in comparison to other physical exercise intervention studies, recruiting similar patients, prior to chemoradiotherapy [mean 43.96, SD 7.47 (76); mean 67, SD 20 (52)]. This pattern continued for the follow up data. Evidence to suggest that physical exercise impacts on QoL in HNC patients remains inconclusive (17).

Findings from the IPAQ suggest that a larger proportion of patients engaged in activity on completion of the intervention compared with baseline. Elsewhere, observational studies have reported a reduction in activity following HNC treatment compared to their baseline status on the same scale (77). Over 80% of the completers reported being involved in at least moderate activity by the end of the programme, a similar figure to those reported by McCarter et al. (78), at 12 months post HNC-treatment. Whether our intervention facilitated earlier, and sustained exercise levels is unknown and warrants further investigation with longer term follow-up.

The physical fitness tests were completed by most patient participants, with the most problematic being the Arm Curl Test due to its proximity in time to surgery and the associated musculoskeletal pain and dysfunction at the flap donor site. There are no alternative upper body muscular strength and endurance tests that avoids this issue. Median assessment results were similar pre- and post-intervention, which can be viewed as a positive response given that exercise interventions typically attenuate the decline in physical and physiological capacities normally observed during cancer treatment rather than enhance them (79). The present feasibility study was not designed to test efficacy, however, and these results should therefore be interpreted with caution.

### Implications for practice

Our findings suggest that the key components of an exercise programme should include personalisation, tailoring and regular support. Patients and staff could be better informed regarding the benefits of physical exercise following a HNC diagnosis. Further support e.g. motivational interviewing and information resources may be beneficial. Treatment side-effects are a major barrier to remaining active and should be managed accordingly. Flexibility in clinical appointments may enable patients to better organise their time to accommodate regular activity. Access to physiotherapists appears variable across HNC centres, disadvantaging a proportion of patients.

### Strengths and weaknesses

This study was a novel, comprehensive mixed methods evaluation addressing multiple uncertainties surrounding the integration of a physical exercise intervention into the HNC usual care pathway. All study objectives were met. Recruitment took place across two centres, providing rich contextual information. Qualitative findings provided in-depth information on programme refinement and care pathway integration, although it is noteworthy that most patients in the qualitative study self-identified as active, and most completed the intervention. ACTIOHN achieved a range of patient demographics and characteristics. However, this may not fully represent the HNC population, where laryngeal cancer rates are higher, and socio-economic status is lower. Furthermore, there was low representation from ethnic minority groups. No participants had complex airway issues. A full understanding of attrition was compounded by fewer interviews or recorded reasons for decliners and non-completers. As patients were recruited across a range of timepoints across their cancer treatment pathway, a sample size calculation to power a trial was not conducted.

### Future research

Several barriers were identified, that could be addressed to expand recruitment, increase retention and adherence, and improve research processes. A high completion rate was achieved for all assessments, although further work is needed to identify the most suitable primary outcome measure. Although all but one patient agreed to participate on the first recruitment approach, there were no signals to indicate whether better outcomes were achieved when the intervention was delivered pre- or post- cancer treatment and requires further investigation. Further input from key stakeholders (patients, relatives, clinicians, service managers, HNC charities and researchers) is required to explore solutions, refine, and co-develop a research protocol ready for a future trial to evaluate effectiveness. Support materials require development e.g. CES training package and its delivery, bespoke patient information. The programme should be tested in a larger number of centres, to study its integration to routine HNC care across the UK, with longer-term follow up.

## Conclusions

People with HNC face unique challenges that can make it harder for them to exercise. Evidence taken from exercise oncology research on other cancer sites cannot simply be extrapolated to HNC. This study suggests that ACTIOHN is a feasible and acceptable intervention, but some adjustments are required to improve acceptability, recruitment processes, retention and adherence. A personalised, remotely delivered approach was valued by patients. Overall, patients were positive about the programme, for both their physical and mental well-being. As the prescribed focus of this study was feasibility and acceptability, no definitive conclusions can be made regarding the benefit of exercise. However, with refinement of the intervention and trial delivery, we believe that a definitive trial would have the ability to quantify the benefits to patients. Further research is required to evaluate short and long-term effectiveness and cost-effectiveness of, and patient engagement with, personalised exercise for HNC survivors.

## Data Availability

The original contributions presented in the study are included in the article/**Supplementary Material**. Further inquiries can be directed to the corresponding author.
