# Supplementary material for: ACTivity as medicine In Oncology for Head and Neck (ACTIOHN): a feasibility study investigating a patient-centred approach to exercise for head and neck cancer patients
Source: Front Oncol. 2025 Jul 11;15:1525512. doi: 10.3389/fonc.2025.1525512 (PMC12289591; doi:10.3389/fonc.2025.1525512)
Supplement: Supplementary file 1 [file Table1.docx]

**Supplementary file 1.** Measurement properties of the pre- and post-intervention assessments. Assessments of physical fitness will be performed in the order shown in the table in accordance with recommendations [31].

| Outcome | Measurement Tool | Attributes |
| --- | --- | --- |
| Fatigue | Multidimensional Fatigue Symptom Inventory - Short Form (MFSI-SF) | Comprises 30 statements or questions with a 5-point Likert-scale scoring response that assesses fatigue across five domains: 1) general fatigue; 2) physical fatigue; 3) emotional fatigue; 4) mental fatigue; and 5) vigour [32]. A total score is obtained by summing the first four subdomains and subtracting Vigor, which has a range from minus 24 to plus 76. |
| Health-related quality of life | Short-Form 36 Health Survey Questionnaire (SF36) | Comprises 36 questions that explore eight domains of health: 1) physical functioning; 2) role limitations due to physical health; 3) role limitations due to emotional problems; 4) energy/fatigue; 5) emotional well-being; 6) social functioning; 7) pain; and 8) general health [33]. |
| Physical activity levels | International Physical Activity Questionnaire (IPAQ) – Short Form | Comprises seven questions that explore the amount of moderate and vigorous physical activity performed and the amount of time spent walking and sitting time over the past 7 days [34]. |
| Lower body muscular strength and endurance | 30-Second Chair Stand Test | Maximum number of times within 30 s that an individual can rise from a seated position to a full stand without pushing off with the arms [31]. ICC = 0.89 [35]. |
| Upper body muscular strength and endurance | 30-Second Arm Curl Test | Maximum number of times a hand weight can be curled through a full range of motion in 30 s. Hand weight is 5 lb for women and 8 lb for men [31]. ICC = 0.81 [35]. |
| Cervical spine and shoulder range of motion | Long-arm goniometer | Cervical spine range of motion in forward flexion, extension, lateral flexion, and rotation. Shoulder range of motion in flexion, abduction, and lateral and medial rotation [36]. |
| Power, speed, agility, and dynamic balance | 8-Foot Up-and-Go Test | Shortest time taken to rise from a seated position on a chair, walk 8 feet around a cone, and return to the chair and sit back down [31]. ICC = 0.95 [35]. |
| Aerobic endurance | 6-Minute Walk Test | Maximum distance that can be walked in 6 minutes along a 30 m course [79]. ICC = 0.94 [35]. |

**ICC** = test-retest intraclass correlation coefficient.

**Supplementary file 2** Example exercise sessions

**Monday**

| Warm-up | Pulse raiser: March on the spot or around house/garden for 5 min, starting at a rating of perceived exercise (RPE) of 9, progressing to RPE 11.  Stretches: Quadriceps, hamstrings, calves. Hold each stretch for 10 sec. |
| --- | --- |
| Main component | Walk for 30-60 min at RPE 11-13 |
| Cool-down | Pulse lowerer: March on the spot or around house/garden for 5 min, starting at an RPE of 11, regressing to RPE 9.  Stretches: Quadriceps, hamstrings, calves. Hold each stretch for 20 sec each leg/side. |

**Tuesday**

| Warm-up | Pulse raiser: March on the spot or around house/garden for 5 min, starting at an RPE of 9 progressing to RPE 11.  Joint mobilisation: Gentle shoulder rolls (forward and backwards) and shrugs. Increase size of the movement with subsequent repetitions.  Stretches: Quadriceps, hamstrings, calves, neck stretches (flexion/extension, side flexion, rotation), shoulder stretch with hands on table, cross body shoulder stretch. Hold each stretch for 10 sec. |
| --- | --- |
| Main component | 1. Bilateral biceps curls with resistance band 2. Shoulder flexion wall slides with towel 3. Shoulder abduction with resistance bands 4. Shoulder internal rotation with resistance band 5. Shoulder external rotation with resistance band 6. Sit to stand (no arms) 7. Seated knee extension with resistance band 8. Heels raises holding onto a chair   1-2 sets of 10-15 reps for each exercise, with 2 min rest between sets |
| Cool-down | Pulse lowerer: March on the spot or around house/garden for 5 min, starting at an RPE of 11 regressing to RPE 9.  Stretches: Quadriceps, hamstrings, calves (hold each stretch for 20 sec), neck stretches (flexion/extension, side flexion, rotation), shoulder stretch with hands on table, cross body shoulder stretch (hold each stretch for 20-30 sec). |

**Supplementary file 3** Progression criteria for feasibility testing

|  | **Green** | **Amber** | **Red** | **Status** | **Implications** |
| --- | --- | --- | --- | --- | --- |
| **Recruitment** | **≥ 33% identified as eligible and approached were recruited** | **At least 20 HaNC patients were recruited** | **< 20 HaNC patients recruited** | **Green: 71% patients consented to the study. 58 (54%) completed the Physio assessments and CES planning meetings.** | **Recruitment rate was achieved. High uptake, but drop out before taking up the intervention.** |
| **Retention** | **Proposed sample size is retained** | **At least 20 HaNC patients were and retained** | **< 20 HaNC patients retained at follow-up** | **Amber: 40 patients were retained** | **Strategy for retaining people who are unwell – temporary pause? CES trained in CVD?** |
| **Data completion** | **> 85% of outcome data collected** | **> 60% of outcome data collected** | **< 60% of outcome data were collected** | **Green: >80% of outcome data were collected** | **High investment from Physiotherapist and RNs. Information took time to upload** |

**Supplementary file 4** Details of patient and public involvement (PPI) in the ACTIOHN study.

| Aim | 1) Develop a study design and an intervention that tackles patients’ concerns  2) Involve patients throughout the whole research cycle |
| --- | --- |
| Methods | VB, a highly experienced PPI representative co-developed the study design and intervention for ACTIOHN.  VB attended all steering group, project advisory group and study launch meetings. VB chairs a HNC PPI group called CHANGE (Cancer of Head And Neck Group Experience). CHANGE comprises six HNC survivors of mixed sex and various ages. The group met before and during the project. CHANGE co-developed patient-facing documents and support materials, advised on approach, consent and recruitment processes, were involved in qualitative analysis and data interpretation. VB was involved in disseminating findings. |
| Results | 1. Study design and intervention development  VB and CHANGE helped to prioritise, develop and refine the research plan. CHANGE confirmed that HNC survivors have unique issues, making exercise difficult. CHANGE felt that fatigue experienced during and after cancer treatment could affect survivors’ willingness or ability to exercise. They said it was important that any exercise programme should be personalised and flexible, finding ways to overcome substantial barriers.  Members were uncertain as to what the best time point might be to introduce physical exercise. This uncertainty was addressed in the project aims and methods, enabling patients to join the intervention pre-, during or post-treatment. CHANGE reviewed the proposed assessments and agreed the level of burden was appropriate. VB co-produced a Plain English Summary and PPI plans.  2. Research cycle  During project set-up, CHANGE advised on recruitment procedures and helped develop patient information. CHANGE were instrumental in deciding on support materials for exercise adherence and overcoming barriers using their personal experiences. Together with our Behavioural Psychologist they produced a ‘top tips’ and ‘exercise stories’ leaflet on managing HNC symptoms to enable engagement with exercise and increase physical activity.  CHANGE provided insights and perspectives on interview transcripts, identifying and prioritising important messages, enabling credibility checks, and helping to direct further probing in subsequent interviews.  VB presented at two conferences about PPI in the study (International HNC Quality of Life Conference 2022 and RED Talk, Liverpool Head and Neck Centre https://livheadandneck.co.uk/), and has co-authored publications from the study. |
| Discussion | PPI was an integral part of the whole study, ensuring the patient voice was heard throughout. PPI influenced key components e.g. the personalisation of the intervention, the need for flexibility, capturing adherence, support materials. As Chair of CHANGE, VB was able to capture a range of views. However, it is acknowledged the group lacks representation from certain groups (ethnicity, low socio-economic status). |
| Reflections | ‘*As PPI co-applicant I felt I was listened to; feedback from CHANGE was acted upon, directing the course of the study. This was evident from before the study even began when we were just kicking ideas around. I never felt as if my ideas and suggestions, and those of CHANGE were less valuable. Trying to expand CHANGE to include a wider range of members is difficult, we are aware of this and are trying to address it. Our next step is to look at what further training is available for PPI representatives, to help with the subsequent project.’* |

**Supplementary file 5:** Questionnaire outcomes completers and non-completers

|  |  | Baseline Completers | | Baseline Non-completers | | Baseline Total | Follow up |
| --- | --- | --- | --- | --- | --- | --- | --- |
| N |  | 39 | | 30 | | 69 | 39 |
| **MFSI** |  | | | |  | | |
| General | Median (IQR)  Range | 5 (12)  (0, 23) | | 9 (11)  (0, 21) | | 6 (11)  (0, 23) | 4.5 (11)  (0, 21) |
| Physical | Median (IQR)  Range | 3 (5)  (0, 16) | | 5 (13)  (0, 18) | | 4 (8)  (0, 18) | 2.5 (6)  (0, 20) |
| Emotional | Median (IQR)  Range | 2 (8)  (0, 18) | | 6 (11)  (0, 21) | | 3 (8)  (0, 21) | 3 (6)  (0, 17) |
| Mental | Median (IQR)  Range | 4 (4)  (0, 12) | | 5 (4)  (0, 17) | | 4.5 (5)  (0, 17) | 2 (5)  (0, 16) |
| Vigor | Median (IQR)  Range | 12 (7)  (2, 24) | | 9.5 (5)  (1, 18) | | 11 (6)  (1, 24) | 15 (9)  (2, 24) |
| Total | Median (IQR)  Range | 4 (24)  (-20, 50) | | 11.5 (43)  (-11, 67) | | 7 (30)  (-20, 67) | 1 (21)  (-19,65) |
| **SF36** |  | | | |  | | |
| Physical functioning | Median (IQR)  Range | 87.5 (41)  (0, 100) | | 72.5 (53)  (0, 100) | | 75 (45)  (0, 100) | 85 (33)  (25, 100) |
| Limitations – physical health | Median (IQR)  Range | 50 (100)  (0, 100) | | 0 (94)  (0, 100) | | 25 (100)  (0, 100) | 50 (100)  (0, 100) |
| Limitations – Emotional | Median (IQR)  Range | 100 (75)  (0, 100) | | 33.33 (100)  (0, 100) | | 100 (100)  (0, 100) | 100 (88)  (0, 100) |
| Energy | Median (IQR)  Range | 60 (35)  (0, 100) | | 47.5 (28)  (0, 90) | | 55 (30)  (0, 100) | 55 (34)  (0, 95) |
| Emotional Well being | Median (IQR)  Range | 80 (23)  (32, 100) | | 68 (28)  (8, 96) | | 76(28)  (8. 100) | 78 (28)  (24, 100) |
| Social functioning | Median (IQR)  Range | 75 (63)  (13, 100) | | 62.5 (63)  (0, 100) | | 68.75 (75)  (0, 100) | 62.5 (50)  (13, 100) |
| Pain | Median (IQR)  Range | 67.5 (45)  (10, 100) | | 45 (58)  (0, 100) | | 57.5 (58)  (0, 100) | 66.25 (33)  (10, 100) |
| General health | Median (IQR)  Range | 70 (25)  (20, 100) | | 65 (23)  (20, 90) | | 65 (20)  (0, 100) | 70 (25)  (5, 90) |
| **Fitness Assessment** | | |  | | | | |
| Chair Stand Test (repetitions) | Median (IQR)  Range | 12 (6)  (2, 27) | | 11.5 (6)  (4, 22) | | 12 (6)  (2, 27) | 13 (7)  (7, 35) |
| Arm Curl Test (repetitions) | Median (IQR)  Range | 20 (10)  (0, 29) | | 14 (16)  (0, 32) | | 15 (13)  (0, 32) | 20 (8)  (6, 31) |
| 8ft Up and Go Test (sec) | Median (IQR)  Range | 7.45 (3.2)  (3.3, 23.9) | | 9.2 (3.8)  (2.9, 18.9) | | 8.21 (3.7)  (2.9, 23.9) | 6.65 (1.8)  (3.4, 11.7) |
| 6 min Walk Test (metres) | Median (IQR)  Range | 325 (243)  (50, 650) | | 260 (254)  (80, 620) | | 300 (255)  (50, 650) | 400 (530)  (260, 658) |
| ***IPAQ*** |  | | | |  | | |
| Vigorous physical exercise | None  Some  Missing | 26 (68.4)  12 (32.6)  2 | | 22 (81.5)  5 (18.5)  2 | | 48 (73.9)  17 (26.1)  4 | 18 (47.4)  20 (52.6)  2 |
| Moderate physical exercise | None  Some  Missing | 13 (34.2)  26 (65.8)  1 | | 18 (69.2)  8 (30.8)  3 | | 31(48.4)  33 (51.6)  4 | 11 (29.0)  27 (71.0)  2 |
| Time spent moderate exercise (hours) | Median (IQR)  Range | 1 (1.5)  (0, 7) | | 1 (5.83)  (0, 8) | | 1(1.58)  (0, 8) | 1.25 (1.1)  (0, 6) |
| Walking (days week) | Median (IQR)  Range | 5 (6)  (0, 7) | | 3.5 (7)  (0, 7) | | 4 (7)  (0, 7) | 7 (4)  (0, 7) |
| Walking (hours per day) | Median (IQR)  Range | 1 (1.5)  (0.17, 8) | | 1 (1.5)  (0.10, 7) | | 1 (1.5)  (0.10, 6) | 0.75 (5)  (2, 6) |
| Sitting (hours per week day) | Median (IQR)  Range | 5 (4)  (1, 16) | | 7.75 (6)  (3, 16) | | 5 (6)  (1, 16) | 6 (5.8)  (2, 15) |
| Activity level | Low  Moderate  High | 11 (50.0)  4 (28.2)  7 (31.8) | | 12 (35.3)  7 (20.0)  15 (44.1) | | 23 (33.3)  11 (15.9)  22 (31.9) | 7 (17.9)  13 (33.3)  19 (48.7) |

**Supplementary file 6:** Summary of MFSI and SF36 change pre to post intervention

| **MFSI** | **Change** | **Frequency** |
| --- | --- | --- |
| General | Decreased  Same  Increased  Missing | 15  2  20  3 |
| Physical | Decreased  Same  Increased  Missing | 15  6  17  3 |
| Emotional | Decreased  Same  Increased  Missing | 14  8  16  2 |
| Mental | Decreased  Same  Increased  Missing | 28  5  6  1 |
| Vigor | Decreased  Same  Increased  Missing | 13  9  17  1 |
| Total | Decreased  Increased  Missing | 18  17  5 |
| **SF36** |  |  |
| Physical functioning | Decreased  Same  Increased  Missing | 12  6  18  4 |
| Limitations – physical health | Decreased  Same  Increased  Missing | 13  15  10  2 |
| Limitations – Emotional | Decreased  Same  Increased  Missing | 7  20  11  2 |
| Energy | Decreased  Same  Increased  Missing | 23  2  14  1 |
| Emotional Well being | Decreased  Same  Increased | 18  4  18 |
| Social functioning | Decreased  Same  Increased  Missing | 18  8  13  1 |
| Pain | Decreased  Same  Increased  Missing | 18  2  19  1 |
| General health | Decreased  Same  Increased  Missing | 16  4  17  3 |

**Supplementary file 7** Summary of shoulder movement change Pre to post intervention

| ***Shoulder*** | **Change** | **Frequency** |
| --- | --- | --- |
| Flexion (L) | Improved  Same  Reduced  At full ROM Pre intervention – no change | 14  2  1  23 |
| Flexion (R) | Improved  Same  Reduced  At full ROM Pre intervention – no change  Missing | 13  2  5  18  2 |
| Abduction (L) | Improved  Same  Reduced  At full ROM Pre intervention – no change | 13  3  3  21 |
| Abduction (R) | Improved  Same  Reduced  At full ROM Pre intervention – no change  Missing | 13  2  6  17  2 |
| Lateral rotation (L) | Improved  Same  Reduced  At full ROM Pre intervention – no change  Missing | 9  22  1  6  2 |
| Lateral rotation (R) | Improved  Same  Reduced  At full ROM Pre intervention – no change  Missing | 6  20  4  6  4 |
| Medial rotation (L) | Improved  Same  Reduced  At full ROM Pre intervention – no change  Missing | 9  22  3  4  2 |
| Medial rotation (R) | Improved  Same  Reduced  At full ROM Pre intervention – no change  Missing | 8  20  4  4  4 |
| ***Cervical*** |  |  |
| Forward Flexion | Improved  Same  Reduced  At full ROM Pre intervention – no change  Missing | 16  18  0  2  4 |
| Extension | Improved  Same  Reduced  At full ROM Pre intervention – no change  Missing | 18  16  1  3  2 |
| Lateral flexion (L) | Improved  Same  Reduced  At full ROM Pre intervention – no change  Missing | 16  17  2  3  2 |
| Lateral flexion (R) | Improved  Same  Reduced  At full ROM Pre intervention – no change  Missing | 11  21  1  3  4 |
| Rotation (L) | Improved  Same  Reduced  At full ROM Pre intervention – no change  Missing | 11  22  2  3  2 |
| Rotation (R) | Improved  Same  Reduced  At full ROM Pre intervention – no change  Missing | 12  26  5  3  4 |

**Supplementary file 8:** Barriers, recommendations and illustrative quotations

| **Barriers** | **Illustrative Quotations** | **Suggested Improvements** |
| --- | --- | --- |
| Inconsistent understanding of the benefits of activity for HNC patients | The clinicians have no knowledge [of the benefits of activity for HNC patients]. And, in fairness, why would they? They’re amazing at what they do, but that’s their role. However, I do feel, if there was a one-day or a two-day CPD, where they could be educated on the importance of nutrition and exercise, they could be having these conversations with the patients during that treatment pathway. HP04_CES_ Involved  Some of the patients that I tend to see who aren't managing enough nutritionally, their energy levels are really low. (…) I think at that point, though, they're probably not medically well enough to be doing any exercise. HP08_SLT_not_involved | - Provide clear explanations of the rationale for the trial, including how activity can promote recovery, to all eligible patients. Allow time for questions to be raised and answered and misconceptions to be clarified - Deliver patient information in an accessible way, perhaps using multiple formats (e.g. video, written) and taking account of prior knowledge and activity levels, health literacy and literacy levels - Ensure patients receive a consistent message from HCPs regarding the value of activity - Ensure that language used when discussing the programme and trial is consistent and appropriate (e.g. activity versus exercise) - Ensure that all HCPs working with HNC patients are fully trained in the benefits and risks of activity so that they can confidently discuss this with patients. |
| Poor understanding of the rationale for, and format of, the trial | Well, it was quite daunting [joining an activity programme] because I did the initial meeting with [Physio] before the programme started, where I did some test exercises. (…) I thought, “Well, this might be a bit more than I was expecting” (…) But when I did those specific tests I thought, “What's he going to have me do here? Because I'm going to be struggling if it’s any more siting and standing and walking any great distances”. P34_Site2_Completed  I wasn’t 100% sure about what exactly it [the study] was intending to achieve, for me, personally, until of course I spoke with [CES], and we drew the actual specific programme up. It was tailored, and I didn’t initially appreciate, with [physio], how tailored it would be. P019_Site1_Completed  What the actual idea was, the exercise and why they think you should do that. I mean I read about that and it says it aids recovery. I actually read that. Nobody actually told me that it would aid recovery. Then I thought, “Well what sort of exercise are you actually going to be giving you to do?” Was it a programme set up to be given to you to see that you do two hours a day or an hour a day or half an hour a day? Nothing like that was explained. It just said an exercise programme [in the participant information sheet]. P09_Site2_Declined |  |
| Difficulties balancing the side effects of treatment with engagement | At that point [when side effects of treatment began], I think motivation was probably, I think, the most difficult. I did force myself to do about three or four of the sessions and they would mainly be the resistance sessions. It was the cardio that I missed out, just because I didn't want to lose too much weight. Yes, I did have force myself. I did have to force myself. P012_Site1_Completed  When I had had my next lot of chemo, which I think was three or four weeks after I had had my first lot, that’s when I was really poorly, and I had to stop the programme. Because I said to her, “No offence. It’s been great.” But I just couldn’t, because it completely wiped me out. And I wasn’t eating either (…) It probably would have worked better [if I had been able to pause the programme]. Because when I was poorly I stopped it. And then when I started feeling better I probably would have said, “I will pick it up again.” P030_Site1_Withdrawn  I, literally, couldn’t get out of bed some days. You know, there was just nothing there. The main thing of my whole existence was to get to my treatment and go through that (…).I think I took one… I think, one week [away from the programme], I took about five days when I said I just couldn’t do anything then. P54_Site2_Completed  When they get to week four, five and six and seven, that’s when they start to get tired. So, I suppose, if they’re feeling tired, their neck is sore, their throat is sore, they’re not eating that well, the last thing on their mind has got to be saying, “Well, I’ve got to get that lazzi band out now. I’ll do a bit of exercise.” They’re not going to want to be bothered, because it’s also they’ve got to go to [hospital], you have the treatment, come back from [hospital], and they only get the weekend off, really, for good behaviour. So, I think, with them it’ll be the fatigue and the time that they’ve got. HP13_CNS_not_involved | - Ensure CESs receive HNC-specific training to allow them to safely deliver the programme to patients undergoing or recovering from acute medical treatment - Refine the timing of the programme and/or permit pauses in the programme to allow patients to gain maximum benefit from it - Ensure patients have timely access to other HCPs as and when needed (e.g. physiotherapists; dieticians) |
| Supporting patients through treatment | I have had to research a lot of stuff myself in terms of certain side effects from the radiotherapy, which I didn't anticipate or I didn't realise would be- So it might have been good to have someone be able to sit and just explain a little bit more detail, the impact of treatment to a patient. …That would have been quite beneficial. (…) Rather than me having to just trawl the internet and try and find more information about it. HP02_CES_ Involved  They didn’t actually get to see the nutritionist until week five. And so some of them had already been struggling, from a nutritional perspective, from week three. So I found it quite interesting that they didn’t have that appointment. Because I was saying, “Have you seen the nutritionist yet?” because I was very conscious that they needed support and guidance. HP04_CES_Involved  We don’t have any physio on the team as part of the MDT team but I think that could be an area for improvement in the pathway. If somebody did have muscle weakness or wanting an exercise programme, if we did refer to the physios they would say there’s an eight-week waiting list, so by the time you’ve referred they’ve already finished their treatment. HP11_Dietician_not_involved |  |
| Engagement with, and acceptability of, patient materials | The participant logbook I thought it's a waste of trees, we've got this really thick book that is just an absolute waste (…) I get that some people may not have access to a laptop, a computer, or an app, or something like that – but give them the option because the amount of paperwork you will save and time and effort for everybody if this could be emailed- And plus then it means it can be shared and viewed electronically as part of the review on a weekly basis with the team. P026_Site2_Completed  The logbooks are very long-winded and they're paper. Whether we could look at making that more easily accessible with either online or making them a little bit shorter. HP01_Physiotherapist_Involved  Physitrack has been a major problem (…) Some patients I had, they didn't even want to see the link, they didn't want anything to do with the link. Straightaway, the link was a barrier for them. HP02_CES_ Involved  One thing that didn’t happen was… [physiotherapist] sent me a link to something, an online something or other, and it didn’t recognise me. I emailed her saying it hadn’t recognised me. Then I tried again and again and it didn’t, so I didn’t use that facility. P020_Site1_Completed | - Consider multiple ways of giving and collecting information from patients (e.g. giving patients a choice of a digital or paper logbook; offering them a choice of the Physitrack app or paper copies of activities) - Consider refining questionnaires to remove duplication, and allow patients opportunity to complete these independently - Explain to patients the rationale for choice of assessment methods |
| Engagement with, and acceptability of, outcome measures | I felt a little bit pressured with that [questionnaire completion] because of doing it while she [physiotherapist] was waiting. I was trying to do it quickly. Yes, and I thought, “Maybe I'm not answering truthfully on some of these questions,” because I felt a little bit under pressure. Yes, so that might be better if you’re just left, maybe, to just fill it in. Yes, that you go and wait in a waiting room or somewhere, to fill it in, yes. P021_Site1_Withdrawn  The assessments felt like- this is in no way demeaning for anybody else, but felt like they were maybe designed for older people. I don't actually know the stats. I don't know if I'm relatively younger than most people who have this diagnosis, but it didn't really test me physically. P012_Site1_Completed |  |
